# New Approaches to Treat Osteoarthritis with Mesenchymal Stem Cells

**DOI:** 10.1155/2018/5373294

**Published:** 2018-09-16

**Authors:** Kiran Shah, Ashley G. Zhao, Huseyin Sumer

**Affiliations:** ^1^Australian Veterinary Stem Cells and Magellan Stem Cells P/L, 116-118 Thames St, Box Hill VIC 3129, Australia; ^2^Department of Chemistry and Biotechnology, Faculty of Science, Engineering and Technology, Swinburne University of Technology, John St, Hawthorn VIC 3122, Australia

## Abstract

Osteoarthritis is one of the most common chronic health problems in the world that causes disability and chronic pain with reduced mobility and is a progressive degenerative disease in weight-bearing joints such as the knee. The pathology of the joint resulting from OA includes loss of cartilage volume and cartilage lesions leading to inflammation of the articular joint structures; its incidence and progression are associated with a variety of risk factors. Most of the current treatments focus on symptom management such as physical and occupational therapies, pharmacological intervention for pain management, and surgical intervention with limited success and do not address nor halt the progression of the disease. In this review, we will describe the current treatment options for OA and the exciting new translational medical research currently underway utilising mesenchymal stem cells for OA therapy.

## 1. Osteoarthritis

**Figure 1:**
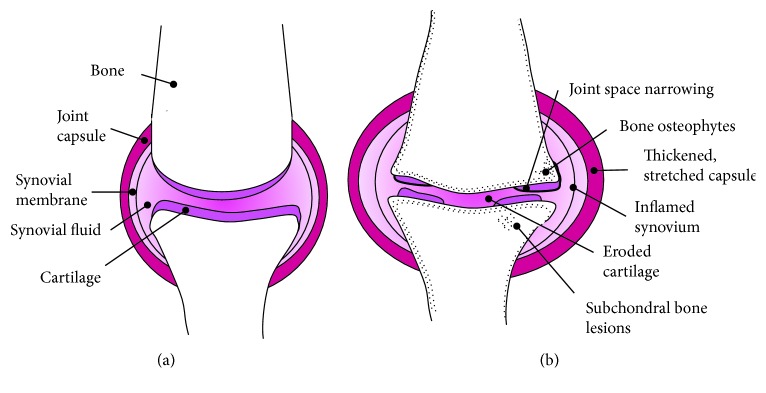
Diagram of the synovial knee joint in (a) a healthy individual and (b) an individual with mild osteoarthritis.

Osteoarthritis is the pathology of articular joints most commonly associated with defects in cartilage such as osteochondral defects and is one of the most common chronic disabling diseases affecting people worldwide. It can cause severe limitation of daily activities that can seriously affect the quality of life. Approximately, 9.6% of men and 18% of women who are over 60 years old have symptomatic osteoarthritis worldwide [1, 2]. The musculoskeletal condition is characterized by degenerative articular cartilage that leads to thinning of cartilage (Figure 1) resulting in bone contact, eventually leading to symptoms of stiffness, pain, and limitation of movement. The major risk factors for OA are older age, obesity, previous injury, sports-related injury, occupational overuse, and genetic background [3, 4]. As the elderly population and obesity increase around the world, OA has become more widely spread causing a substantial health and economic burden globally [5]. It is estimated that associated costs of OA have a socioeconomic burden between 1.0 and 2.5% of gross domestic product in developed countries [6].

OA is usually associated with synovial joints (diarthroses), also known as the freely moveable joints [7]. The normal synovial joint (Figure 1) is formed by two bones' ends covered with a thin layer of smooth, firm articular cartilage, a capsule filled with the synovial fluid, ligaments, tendons, muscles, blood vessels, and nerves [8]. Those structural components form a functional unit with their mechanical interaction. The changes in any component lead to the anabolic or catabolic responses in other components [9]. The abnormality in the synovial joint tissues such as articular cartilage, subchondral bone, ligaments, menisci, synovium, peripheral nerves, and muscles can cause stress in the joint and eventually result in degeneration of articular cartilage resulting in OA [7, 10].

Articular cartilage is a special type of connective tissue which is nonneural, nonlymphatic, nonvascular, and therefore restricted in self-repair. Articular cartilage is a metabolically active tissue, and its architecture and biochemical composition are regulated, developed, and repaired by chondrocytes. Chondrocytes are the only cell type in the articular cartilage [9]. Nutrition is supported by the synovial fluid and subchondral bone by diffusion through regular joint movement. The movement of the synovial joint forces the synovial fluid in and out of the articular cartilage to deliver nutrients and dispose of waste products for cartilage [11]. The proximal subchondral bone provides nutrients such as glucose, oxygen, and water to cartilage by perfusion from their dense vessels in the subchondral region [12]. Therefore, cartilage, subchondral bone, and synovium interact with each other and play key roles in pathogenesis of OA when there are abnormal mechanics involving the entire articular joint [13, 14].

OA is also associated with the physiological imbalance of degradation and synthesis by chondrocytes resulting in alterations in the composition of the cartilage matrix [15]. In the early stages of OA, the quiescent chondrocytes become activated to remodel the contents of the cartilage matrix [16], the water content increases, and loss of glycosaminoglycan in the cartilage leads to the changes in cartilage mechanical properties at this hypertrophic anabolic phase [17, 18]. After failure of these early compensating attempts, chondrocytes become catabolic and undergo senescence and apoptosis and ultimately result in the progressive degeneration of articular cartilage [19] which is considered as an irreversible state of OA [18, 20]. Furthermore, fibrillations (microscopic cracks) in the superficial zone are formed, as well as deep fissures, bone marrow lesions, and delamination in the cartilage [21]. In addition to the progressive degraded articular cartilage, subchondral bone interacts with cartilage through various signaling mechanisms that are presented and associated with the increased pain and dysfunction [18], due to peripheral and central pain sensitization [16].

## 2. Current Pharmacological Treatment of Osteoarthritis and Its Limitations

**Figure 2:**
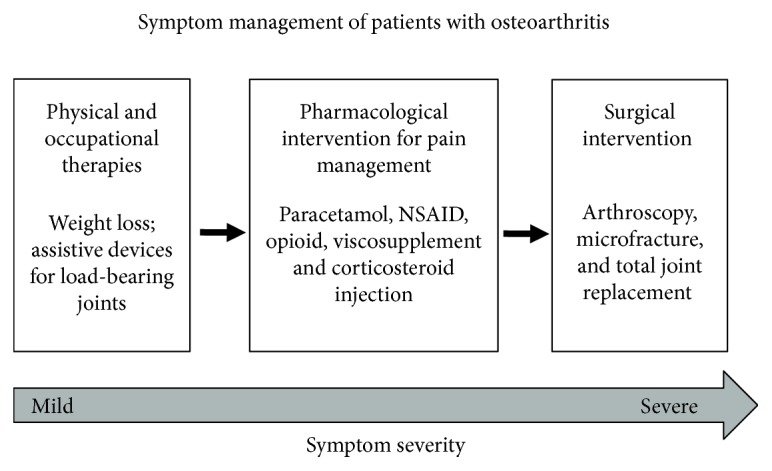
Current recommended treatment modalities for osteoarthritis (source: Arden et al. 2014).

As mentioned above, the degeneration of the articular cartilage remains the most significant structural change seen in OA, resulting in severe pain and reduced mobility [16, 22]. The innate ability to heal the degenerated cartilage is limited by the avascular nature of cartilage, posing a significant challenge in the treatment of OA. Currently, there is no cure for this debilitating condition and most of the treatments focus on the symptom management including 3 main modalities as outlined in Figure 2 [23]. These are, firstly, physical and occupational therapies such as weight loss or assistive devices for load-bearing joints; secondly, pharmacological intervention for pain management by nonsteroidal anti-inflammatory drugs (NSAIDs), opioids, viscosupplements, or corticosteroid injection; and thirdly, surgical intervention such as arthroscopy, microfracture, or total joint replacement.

The Arthritis Foundation (www.arthitis.org) recommends that OA patients undertake self-education in managing the condition and encourages losing weight for overweight and obese patients. This entails diet and exercise to reduce and manage healthy weight; however, due to pain and physical limitation resulting from OA, exercise is hard to implement and sustain. Joint targeted physical therapy has shown to improve the pain and function; however, there is no long-term improvement. An assistive device is designed to provide mechanical support to the joint structure in the patients with OA causing instabilities in the joint and also to distribute the load bearing for relief in pain and improve function, but these only have limited success.

Currently, the primary strategy of OA pharmacological management is mainly to relieve pain, improve function, and manage the OA process [22, 23]. Pharmacological treatment is used for patients with mild to moderate pain, and medications such as NSAID, opioids, and corticosteroid are used routinely to alleviate the pain; however, there is no long-term relief and these pharmacological agents have unwanted side effects [24].

Acetaminophen (paracetamol) used to be the first-line pharmacologic management to treat mild to moderate OA pain. However, it became an inconclusive recommendation due to lack of compelling evidence [15]. Furthermore, using acetaminophen was associated with risks such as gastrointestinal (GI) adverse events and multiorgan failure [17] with minimal short-term benefit [21]. Despite it being less effective than NSAIDs and since some patients have adverse effects with NSAIDs, it is still used by some patients but is recommended with conservative doses and treatment duration [15].

Nonsteroidal anti-inflammatory drugs (NSAIDs) are a big family of drugs including oral NSAIDs such as ibuprofen, aspirin, naproxen, COX-2 inhibitors, and topical NSAIDs such as diclofenac formed as cream, patches, gels, or solution. Issues with oral NSAIDs include adverse gastrointestinal (GI) effects and need to be taken in conjunction with the GI protectant [25]. Furthermore, they are associated with potential toxicity especially in elderly patients [22]. The oral COX-2 inhibitors can reduce GI side effects but can cause other adverse effects such as the risk of cardiovascular events. The use of topical NSAIDs eliminates the GI side effects of the oral NSAIDs but can be less effective [26] and has been associated with dermatological adverse events [27].

Opioids can be used for pain relief when patients cannot use NSAIDs and acetaminophen due to their associated side effects. However, they have limited long-term efficacy [27] and are associated with adverse effects such as respiratory depression, opioid use disorder, and overdose [28]. In meta-analysis of trials, patients who received opioid therapy were four times more likely to drop out due to adverse effects as compared to patients receiving placebo, and their long-term use is not recommended [27]. There is a wide range of medicines aimed at pain relief and improvement of quality of life for patients with OA. However, currently there are no pharmacological agents that can prevent, halt, or reverse the onset of OA. These studies highlight lack of effective pharmacological solutions for OA sufferers.

## 3. Surgical Intervention to Treat OA

Surgical interventions are recommended when the progression of OA has resulted in severe damage to the joint, severity in pain, and function deterioration that cannot be managed with any other options. The initial surgical option to restore the structural stability such as joint debridement by arthrotomy or arthroscopy to remove loose cartilage, fragments of meniscus, shaving of the cartilage, and removing osteophytes has shown to result in limited pain and function relief [29]. Arthroscopy remains the most performed surgery in the developed world by orthopedic surgeons to help with the mechanical movement of the affected stiff knee. A blinded controlled clinical trial on the arthroscopy for the debridement and lavage with a placebo showed that there is no pain relief achieved after the surgery when compared with the placebo [30].

Joint replacement is considered the final option provided to OA patients when the condition progresses to the most severe. Surgical procedures for the replacement of the hip and the knees are extremely painful and require a long period of time for rehabilitation. Furthermore, total knee replacement has shown adverse outcomes such as pulmonary embolism, infections, and surgery-related deaths in some cases [31].

## 4. Cellular Therapy and Regenerative Medicine for OA

In more recent times, many regenerative techniques have been used such as autologous chondrocyte transplantation (ACT) for focal damage of cartilage, microfracture, and mosaicplasty. The ACT technique in addressing confined cartilage damage involves the transplantation of chondrocytes that are harvested from non-weight-bearing cartilage from the patient [32] but does not address generalized OA. This method has some concerns since it causes not only donor site morbidity but also chondrocyte dedifferentiation in the transplanted site leading to the expression of type I collagen rather than type II collagen that may result in fibrocartilage rather than the desired hyaline-like cartilage [33]. Another common surgical technique is the microfracture, which triggers the migration of bone marrow cells to the articular surface through stimulation of inflammatory response by drilling holes in the subchondral plate at the chondral defect site. The purpose of this technique is to provide an enriched environment for tissue regeneration [34]. However, the resultant tissue is again fibrocartilage containing type I collagen or hybrid repair cartilage tissue, not the normal hyaline cartilage (type II collagen). Furthermore, the observed subchondral bone overgrowth (25%–49%) might limit durability and the long-term outcome of the microfracture [35]. Finally, the mosaicplasty procedure is similar to the ACT technique and involves the use of autologous osteochondral grafts; however, the results are disappointingly minimal and only offer short-term benefits [19].

The above demonstrates that the current treatment for OA is only focused on symptom management and none of these options addresses or halts the progression of the disease or offers long-term benefits. Hence, there is an unmet medical demand for the treatment for OA suffers that can halt the progression of the disease and to provide long-term relief from the symptoms of OA. Cellular therapy has provided a real promise to combat this debilitating degenerative condition and can provide disease-modifying long-term benefit. Tremendous efforts have been made in the preclinical studies and now in the clinical trials evaluating the regenerative potential of the adult stem cells, especially mesenchymal stem cells (MSCs), to repair the structural damages of the joint space, cartilage degeneration, and inflammation.

## 5. Mesenchymal Stem Cell Therapy for OA: A New Therapeutic Paradigm for OA

**Figure 3:**
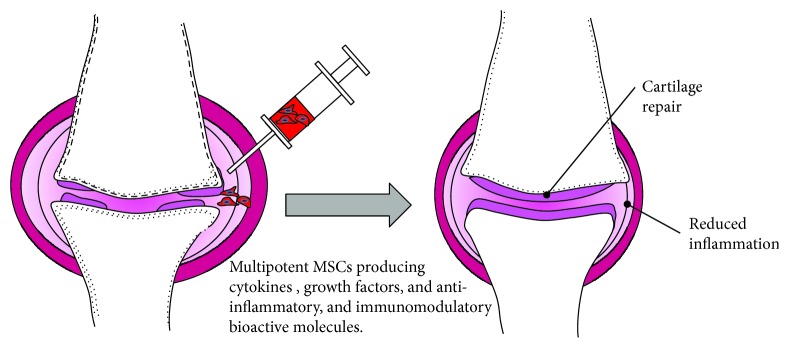
Model of regeneration of a knee joint before and after treatment with MSCs.

Modern medicine is exploring the regenerative potential of cellular therapy to address the currently unmet medical needs of various degenerative conditions such as OA. Cellular therapy has been extensively invested in exploring a new paradigm for the treatment of many degenerative conditions including degenerative disc disease (DDD) and osteoarthritis of the joints, among many other conditions. For this reason, this review will focus on the therapeutic properties of MSCs to treat OA. The disease-modifying potential of cellular therapy such as the use of adult stem cells for regeneration of the damaged tissues has been hailed as a breakthrough in the 21^st^ century and provides an exciting promise to chronic degenerative conditions. Currently, there are over 500 clinical trials registered on ClinicalTrials.gov, exploring the safety and efficacy of adult stem cells, e.g., pluripotent stem cells, umbilical cord-derived stem cells, placental stem cells, and mesenchymal stem cells, to treat OA. Of these, mesenchymal stem cells have been a leading choice for many medical researchers around the world with over 352 registered clinical trials [36]. In the clinical studies, MSCs are isolated from the patient either from the bone marrow or from adipose tissues, purified, and administered as intra-articular injection in the affected joint under ultrasound guidance (Figure 3). MSCs are described to exert their therapeutic effects by homing to the injured site when injected locally to the joint for a short period of time and then disappearing and are believed to be secreting a myriad of growth factors and cytokines to initiate the repair process, as discussed below.

In 1974, Friedenstein and colleagues first described stromal precursors derived from the bone marrow that were able to form plastic-adherent fibroblast colonies in the monolayer culture and their differentiation characteristics [37]. The term mesenchymal stem cell has been in use since it was firstly coined by Caplan in 1991 [38]. MSCs are ubiquitous throughout the musculoskeletal system in the human body and are classified as self-renewing, postnatal, multipotent stem cells that can be differentiated into all tissue types of skeletal system and connective tissues such as bone, fat, cartilage, and muscle [39]. MSCs produce a vast array of cytokines, growth factors, and anti-inflammatory bioactive molecules [40]. MSCs are heterogeneous, clonogenic, and relatively easily isolated from various tissues and can be cultured expended *in vitro* due to their plastic adherence property and have a fibroblast-like morphology under the microscope [41]. Multipotent MSCs are originally derived from the embryonic tissue-mesenchyme which is developed from the mesoderm and can be isolated from various sources including bone marrow, periosteum, trabecular bone, adipose tissue, synovium, skeletal tissues, and deciduous teeth [39]. *In vivo*, the main role of MSCs is believed to be for self-repair and for maintaining tissue homeostasis [42]. The resident MSCs are distributed into the tissues at various stages of maturation and are involved in tissue regeneration [43].

Originally, MSCs were isolated from bone marrow, but more recently they have been successfully isolated from various other tissues such as adipose tissue [44], brain, muscle tissue [45], skin [46], and teeth [47]. Moreover, MSCs can also be derived from different organs and tissues including the spleen, liver, kidney, lung, thymus, pancreas, and blood vessels and could readily be proliferated *in vitro* [48].

Since human MSCs are heterogeneous and can be obtained from many sources, different methods of isolation and expansion, and different approaches to characterize the cells have been described in the literature, this has caused the difficulty of comparing study outcomes. The Mesenchymal Stem Cell Committee of the International Society for Cellular Therapy (ISCT) has provided three minimal criteria to define MSCs for laboratory-based investigation and preclinical studies in 2006. First, MSCs must be plastic-adherent in the tissue culture flasks. Second, more than 95% of the MSC population must express CD105, CD73, and CD90 and lack expression (less than 2% population) of CD45, CD34, CD14 or CD11b, CD79a or CD19, and HLA class II. Third, MSCs must be able to differentiate into osteoblasts, adipocytes, and chondroblasts *in vitro* with standard differentiation conditions [49].

The “stemness” of MSCs is maintained by the leukemia inhibitory factor (LIF), fibroblast growth factors (FGFs), and mammalian homologues of *Drosophila* wingless (Wnts), among other growth factors and cytokines [50]. The intrapopulations of MSCs are functionally heterogeneous regarding their multilineage differentiation potentials. The tripotent clones of MSCs (able to be differentiated into three cell types, e.g., osteoblasts, adipocytes, and chondroblasts) display the highest rate of proliferation and a lower rate of apoptosis compared with the bipotent (only two cell types) and unipotent (only one cell type) clones [51, 52]. The proliferation capacity of MSCs is both affected by the cell seeding density [53] and decreases as cells progress toward terminal differentiations [54]. The long-term expansion of MSCs might impact the composition, function, and therapeutic potency of MSC populations [55]. Furthermore, culture conditions such as culture media and oxygen tension have a major impact on gene expression and proteome and cellular organization [56, 57].

The differentiation process of MSCs is tightly controlled and involves the activities of various transcription factors, cytokines, growth factors, and extracellular matrix molecules [54]. The differentiation efficiency is also correlated with patients' age, whereby isolated cells from younger patients showed higher differentiation capacity in culture [58]. A number of biomarkers are used to determine differentiation towards adipogenic, chondrogenic, and osteogenic lineages. The biomarkers for adipogenic differentiation are adiponectin, C/EBPα, FABP4, leptin, and peroxisome proliferate receptor gamma (PPAR*γ*); the biomarkers for chondrogenic differentiation are aggrecan collagen type II and Sox9; and alkaline phosphatase, bone sialoprotein, osteocalcin, osterix, and runx2 are biomarkers for osteogenic differentiation [59–62].

MSCs have shown disease-modifying effects in bone and cartilage defects, as discussed previously. Because of the multipotent properties of MSCs, they have also generated significant clinical interest in cardiovascular, neural, and orthopedic therapeutic applications. Moreover, the anti-inflammatory and antifibrotic properties of MSCs make them the ideal candidate for regenerative medicine. These cells are able to suppress the growth of activated T-cells and help regulate the production of regulatory T-cells (Treg) [36, 63]. The investigation of the anti-inflammatory properties of MSCs is well advanced, and there are a number of advanced-phase clinical trials for the treatment of graft versus host diseases (GVHD) and Crohn's disease [36]. Furthermore, the therapeutic effects of MSCs have been studied extensively focusing on the immunomodulatory properties and the paracrine activity by secreting a wide variety of cytokines and growth factors that are attributed to the angiogenic and regenerative potential in the damaged tissues [36]. More recently, studies have shown that MSC paracrine effects are mediated by secretion of extracellular vesicles such as exosomes [64, 65]. The use of MSC exosomes might serve as an alternative therapy over MSC transplantation for tissue regeneration [66]. A recent study reported the efficacy of MSC exosomes secreted from the synovial membrane and induced pluripotent stem cell-derived MSCs to treat mouse osteoarthritis whereby both source exosomes demonstrated exosome-attenuated OA [67].

MSC-based treatment of OA has a lower risk to the patient and a variety of sources such as adipose tissue, bone marrow, and synovium [68]. These autologous cells can be harvested from patients by either liposuction or aspirated from bone marrow. The adipose-derived MSCs are preferred by patients as compared to MSCs aspirated from the bone marrow because comparatively MSCs are more abundant in adipose tissues than in the bone marrow aspirate. However, bone marrow-derived MSCs may have higher chondrogenic potential than adipose-derived MSCs [69]. Furthermore, synovial tissues obtained from the surgical removal of subsynovial tissue, a noncartilaginous area of the medial condyle of the femur, have also become an attractive source of MSCs in treatment of OA [9, 68].

## 6. Current Clinical Trials and Case Series Investigating MSCs to Treat OA

A proof-of-concept clinical trial conducted in Korea showed promising safety and efficacy results of adipose-derived MSCs to treat OA. Patients showed reduced pain, improved function of the joint and in the high-dose patient cohort, and regeneration of hyaline-like cartilage suggesting the disease-modifying effects of MSCs when injected into the affected joint [70]. Another pilot study by Orozco et al. demonstrated significant improvement in the pain and functional improvement of up to 65% to 78% in chronic OA patients when treated with bone marrow-derived MSCs, as compared with the conventional treatment methods [71]. Cartilage mapping by T2 MRI showed evidence of improvement in the good cartilage quality, i.e., hyaline-like cartilage, and significant decrease in the poor cartilage quality, i.e., fibrocartilage. The same group conducted a pilot clinical trial examining the safety and efficacy of MSC as a novel treatment of intervertebral disc disorder [72]. After a 1-year follow-up, the primary end point of pain and functional improvement was met in approximately 85% of the cases and no adverse event was observed; the water content was significantly improved in the treated disc and patient-reported significant improvement in the quality of life index [72]. Furthermore, the phase I dose-escalation trial to treat severe OA of the knee by using adipose-derived MSCs to treat patients with symptomatic and severe OA of the knee with single-articular injection of autologous adipose-derived MSCs also showed significant improvement in patients after six months of follow-up [73]. These results showed that the treatment was safe and well tolerated by all patients.

**Figure 4:**
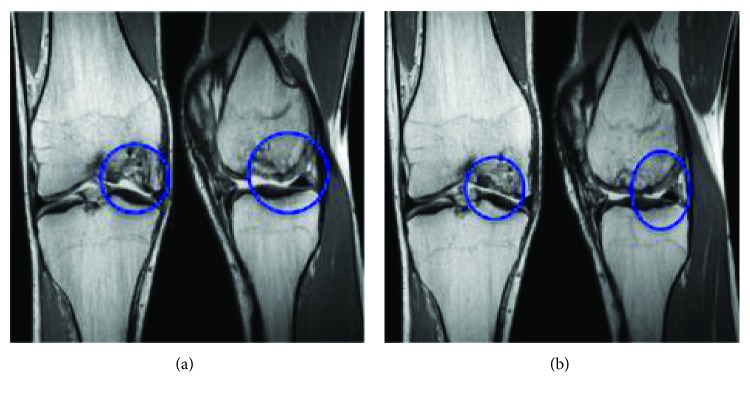
(a) Pretreatment proton density- (PD-) weighted coronal and sagittal MRI images of the knee showing the isolated chondral defect involving the central weight-bearing area of the medial femoral condyle. (b) Posttreatment PD-weighted coronal and sagittal MRI imaging at 18 months indicating articular cartilage regeneration at the site of the osteochondral defect [74].

Adipose-derived MSCs to treat patients with joint disease also act as a precursor to treat degenerative OA. Osteochondritis dissecans is a joint disorder pertaining to articular cartilage and chondral defects resulting in damage to the articular cartilage and underlying bone. Adipose-derived MSCs have been reported to have disease-modifying effects in a clinical case series published recently [74]. This study showed regeneration of the lost cartilage and significant reduction of pain and improvement in mobility (Figure 4) [74].

**Figure 5:**
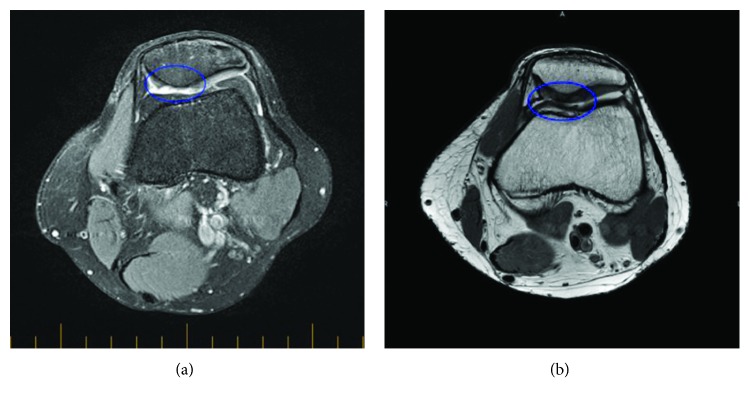
(a) Pretreatment proton density fat-suppressed axial MRI of the knee showing the isolated chondral defect involving the medial facet of the patella. (b) Posttreatment proton density axial MRI indicating articular cartilage regeneration at the site of the chondral defect with smooth integration with the surrounding joint surface [75].

These evidence-based clinical outcomes strengthen the model for treatment of OA with MSCs (Figures 4 and 5). The results of these trials provide an exciting and promising long-term relief for OA patients and herald a new paradigm for the treatment of chronic and debilitating OA and other degenerative conditions. Intriguingly, several hundred clinical trials globally have been registered in the past 10 years, but only a handful of results from these trials are published. Therefore, there is a need for more clinical trial data to be released from the completed trials to further support and develop this novel model of treatment.

## 7. Autologous versus Allogeneic MSCs for Therapy

The choice between autologous and allogeneic MSC treatment is another aspect that will need further supportive data. Due to the immune-privileged aspect of MSCs [63], allogeneic stem cell treatment shows more promise and is likely to attract more attention as an “off the shelf” product. However, long-term safety and efficacy data are warranted. The mechanism involved in modulating the host immune system is believed to be facilitated by the ability of MSCs to influence immune cells' cytokine secretion. MSCs influence mature dendritic type 1 cells to decrease secretion of tissue necrosis factor-alpha (TNF-α) and instruct mature type 2 dendritic cells to increase the anti-inflammatory cytokine IL-10. MSCs can direct T helper cells to decrease secretion of interferon gamma (IFN-*γ*), and T helper cell 2 to increase IL-4 production and help reduce production of IFN-*γ* from natural killer (NK) cells [76–78]. When cocultured with immune cells, MSCs also enhanced the production of prostaglandin E2 (PGE2); therefore, MSCs are able to modulate the immune system by alteration of cytokine production in the host [76–78]. In a comparative study with autologous and allogeneic MSCs, in which 5 patients each received MSCs, the results revealed a similar level of favourable benefits to the quality-of-life improvement in patients with ischemic cardiomyopathy and no immune rejection in the allogeneic group [79]. In a canine study, when autologous and allogeneic MSC transplants were compared in spinal cord injury, both types of cells exhibited therapeutic benefits and transplanted cells were observed in the injured tissue for up to 4 weeks and no immune reactions or adverse effects were reported [80]. Given the safety reports of allogeneic MSC therapy and the surgery-related complications involved in autologous treatment, MSCs derived from a donor for allogeneic therapy provide a better and more affordable treatment option.

## 8. Future Direction

With the approval of Prochymal, an adult stem cell therapy to treat graft versus host disease (GVHD) in children, in Canada and New Zealand, it heralds a new era for cellular therapy to address the unmet medical conditions of previously untreatable diseases. The translational medical research currently underway targeting MSCs for OA therapy in the clinical trial database is promising; however, they need careful evaluation of the outcome data. The results require focus primarily on the safety and then on the efficacy. Furthermore, the various stages of clinical trials currently registered need their outcome data published for the wider scientific community to consider and to evaluate the robustness of the therapy. The large number of MSCs trials indicates the promise of these cells; however, there is considerable paucity of the published clinical trial data and therefore it is early to envisage the extent of their therapeutic application.
